# *C. trachomatis* in Female Reproductive Tract Infections and RFLP-Based Genotyping: A 16-Year Study from a Tertiary Care Hospital

**DOI:** 10.1155/2011/548219

**Published:** 2011-06-26

**Authors:** Satpathy Gita, Mittal Suneeta, Sharma Anjana, Nayak Niranjan, Mohanty Sujata, R. M. Pandey

**Affiliations:** ^1^Department of Ocular Microbiology, Dr R. P. Centre for Ophthalmic Sciences, All India Institute of Medical Sciences, Ansari Nagar, New Delhi 110029, India; ^2^Department of Obstetrics and Gynaecology, All India Institute of Medical Sciences, Ansari Nagar, New Delhi 110029, India; ^3^Stem Cell Facility, All India Institute of Medical Sciences, Ansari Nagar, New Delhi 110029, India; ^4^Department of Biostatistics, All India Institute of Medical Sciences, Ansari Nagar, New Delhi 110029, India

## Abstract

Presence of *Chlamydia trachomatis* in endocervix was determined in 2466 women attending a tertiary care hospital in New Delhi, India over a period of 16 years, using a monoclonal-based direct immunofluorescence assay, tissue culture isolation, and a conventional PCR assay. *Chlamydia* antigen could be detected in 391 out of 2466 (15.85%) of patients studied; in 27.27% women with PID, 16.74% women with cervicitis, 16.03% women with infertility, and 12.06% women with adverse pregnancy outcomes, respectively. There was a statistically significant decreasing trend in *Chlamydia* antigen positivity between the years 1994–1999 and 2000–2004; the apparent decline in antigen positivity between the years 2000–2004 and 2005–2010 was not statistically significant. Antigen detection assay detected equal number of positives as the PCR assay; tissue culture isolation demonstrated lower positivity. In a few representative specimens from cervicitis patients, genotyping was done using RFLP pattern analysis of *C. trachomatis* MOMP gene amplified by PCR assay, all of these belonged to *Chlamydia trachomatis* serovar E.

## 1. Introduction

*Chlamydia trachomatis* was recognized as an important sexually transmitted pathogen after 1970 [1]. Genital *C. trachomatis* infections have emerged as the most prevalent sexually transmitted diseases of bacterial origin [2]. In women, the infection usually manifests as urethritis, cervicitis, salpingitis, and endometritis though a large proportion of the women remain asymptomatic. If untreated, complications and sequelae such as pelvic inflammatory disease (PID), ectopic pregnancy and tubal infertility, and still birth with important socioeconomic consequences are common [3].

To initiate early and appropriate therapy, definitive laboratory diagnosis should be available to the physicians as soon as possible. The physicians should also be aware of the prevalence of infection in their patients to arrive at a quick presumptive diagnosis, since most of the patients remain asymptomatic. Previous studies from different parts of the world, documented that 12–33% of women attending STD clinics and the gynecologist's clinics with cervicitis harbored *Chlamydia* in their cervices [4–6]. In India the prevalence reported from a small study was 10.5% in women in the age group of 20–30 years in Southern India in 2009 [7]. An earlier single point study in 1993 from New Delhi, India reported that 41% of women with vaginal discharge (cervicitis) and 36% of women with infertility were *C. trachomatis* positive [8]. However no long-term study spanning several years regarding *C. trachomatis* involvement in female genital infections has been reported from India. 

*C. trachomatis* consists of 18 serovars, A-K, L_1_-L_3_, D_a_, I_a_, and L_2a_; serovars D-K and L_1_-L_3_ are implicated in genital infections. Determination of causative *C. trachomatis *serovar by conventional methods is cumbersome and usually difficult as the serovar-specific antibodies are not commercially available. The polymerase chain reaction (PCR) assay along with restriction fragment length polymorphism (RFLP) analysis of the major outer membrane protein (MOMP) coding gene was successfully used for genotyping of *C. trachomatis* from clinical specimens [9, 10]. Scanty information is available regarding the prevalent serovars or genotypes of *C. trachomatis* causing infections from India. 

We are regularly testing for the presence of *C. trachomatis* infection in women of child-bearing age coming to the gyneaecology clinic of our hospital for the last 16 years. Furthermore, we genotyped few of the *C. trachomatis* from cervical specimens using RFLP pattern analysis of the *major* outer membrane protein (MOMP) coding gene of *C. trachomatis* amplified by PCR assay.

## 2. Materials and Methods

### 2.1. Study Population

From 1994 to 2010, a total of 2466 women in the age group of 20–50 years clinically suspected of having *C. trachomatis*-related infections and attending the Obstetrics and Gynecology Clinic of All India Institute of Medical Sciences, New Delhi, were included in this study. These comprised of 436 cases of cervicitis, 1671 cases of infertility, 44 cases of pelvic inflammatory disease (PID), and 315 cases with adverse pregnancy outcomes (BOH). 

### 2.2. Specimen Collection

After informed consent, endocervical swabs were collected using a sterile cotton wool swab from the endocervical canal by rotating the swab vigorously for 16–30 seconds. Three separate swabs were collected randomly; one was smeared onto the well of a Teflon-coated glass slide for direct fluorescent antibody (DFA) test, and the other two were placed in vials containing 0.2 M sucrose phosphate buffer (0.2 MSP) medium and transported to the laboratory on ice immediately for PCR assay and tissue culture isolation.

### 2.3. Direct Immunofluorescence Assay (DFA) for Antigen Detection

All the 2466 specimens were processed for* Chlamydia* antigen detection. *Chlamydia trachomatis* direct specimen kit (MicroTrak, USA) was used for the purpose according to the manufacturer's instructions as described before [11, 12]. Briefly, the smears were fixed with cold methanol and stained with fluorescent tagged anti-*Chlamydia *monoclonal antibody (Syva Microtrack, USA) for 30 minutes at 37°C in a humid chamber. The slides were washed with phosphate buffer saline for 10 minutes followed by double distilled water for 5 minutes, air-dried, mounted, and observed under the fluorescent microscope (Nikon, Japan). The positive and negative control sides provided by the manufacturers (Syva Microtrack, USA) were included in each batch of the test.

### 2.4. Isolation of *C. trachomatis* in Tissue Culture

Tissue culture isolation of *C. trachomatis* was done from 1507 of the specimens. The endocervical swabs collected in 0.2 MSP buffer were inoculated onto Mitomycin-C-treated confluent monolayers of McCoy cells grown on cover slips in shell vials via centrifugation at 2000 rpm, according to the method described previously [13]. The inoculated monolayers were incubated at 35°C for 72 hours. The cover slips were taken out, washed with PBS and stained with FITC tagged anti-*Chalmydia *monoclonal antibodies according to manufacturer's instructions using *Chlamydia trachomatis* culture confirmation test kit (MicroTrak, USA). The cover slips were mounted and observed under the fluorescent microscope (Nikon, Japan) for *C. trachomatis* inclusions in Mc Coy cells. Appropriate positive control {*C. trachomatis* L_2_ 434 Bu strain} and negative control (0.2 MSP buffer) were included in each batch of the test.

### 2.5. Diagnostic PCR Assay for Amplification of 517bp Region of *C. trachomatis* Cryptic Plasmid

An in-house conventional PCR assay for amplification of a 517 bp region of *C. trachomatis* cryptic plasmid was used in 333 endocervical specimens from patients with cervicitis using a set of published primers [14], as per the method used by us previously [12]. DNA extracted from *C. trachomatis* L_2_ (434 Bu strain) grown in the yolk sac of embryonated hen's egg and purified by renograffin gradient centrifugation was used as positive control and distilled water was used as negative control. Adequate precautions were taken for avoiding contamination such as use of separate laboratory rooms for DNA extraction, PCR assay procedure and handling of PCR products and use of proper micropipettes. As described previously [12], results were confirmed by southern hybridization with a radio-labeled internal probe.

### 2.6. Restriction Fragment Length Pattern Analysis of the Amplified MOMP Gene for Genotyping

For genotyping using RFLP pattern analysis, the entire *omp1* gene coding for major outer membrane protein (MOMP) was amplified by conventional PCR assay using a set of published primers and procedures described by Sayada et al. [15] from 50 endocervical specimens from patients of cervicitis. A few conjunctival specimens from clinically proven trachoma cases were included for comparison purposes. 

Briefly, DNA was isolated from 500 *μ*L of thoroughly mixed clinical specimens using proteinase K and phenol chloroform extraction as was described before [12]. DNA extracted from *C. trachomatis* L_2_ (434 Bu strain) grown in the yolk sac of embryonated hen's egg and purified by renograffin gradient centrifugation was used as positive control and distilled water was used as negative control. 

PCR assay for amplification of ~1 kb MOMP gene was done in 100 *μ*L volume as was described previously [12]. Ten *μ*L of the PCR amplified product was electrophoresed through a 1% agarose gel to confirm the correct amplification product. The precipitated and purified DNA from the remaining 90 *μ*L of PCR product was used for RFLP analysis with restriction enzyme *Alu *I (NEB, UK), was prepared in a 20 *μ*L reaction mixture containing 3 *μ*g of purified DNA, 15 U of *Alu *I enzyme, and 2 *μ*L of 10x buffer provided with the enzyme and mili Q water *qs*, and was incubated overnight at 37°C. After inactivation of the enzyme at 65°C for 10 minutes, the product was electrophoresed through a 3% agarose gel in an electrophoresis apparatus (LKB, Pharmacia, Sweden), stained with ethidium bromide and visualized under an UV transilluminator. (UVP, UK). In order to attribute clear-cut specificity to the RFLP pattern analysis we included a few conjunctival specimens from which the MOMP gene was PCR-amplified, restriction-digested and the product of this assay was electrophoresed in the same gel.

### 2.7. Data Analysis

STATA 11.0 was utilized for data analysis. Data were systematically recorded and managed on an excel spread sheet. For comparing the proportion positivity amongst the various disease groups such as cervicitis, infertility, PID and BOH, during different time periods, a Pearson *χ* ^2^ test was used. Also *χ* ^2^ test for trend was employed to determine the significant trend during the period. To assess the relationship of DFA with tissue culture, we compared the sensitivity, specificity, positive predictive value (PPV), negative predictive value (NPV), and agreement and their 90% confidence interval values.

## 3. Results

**Figure 1:**
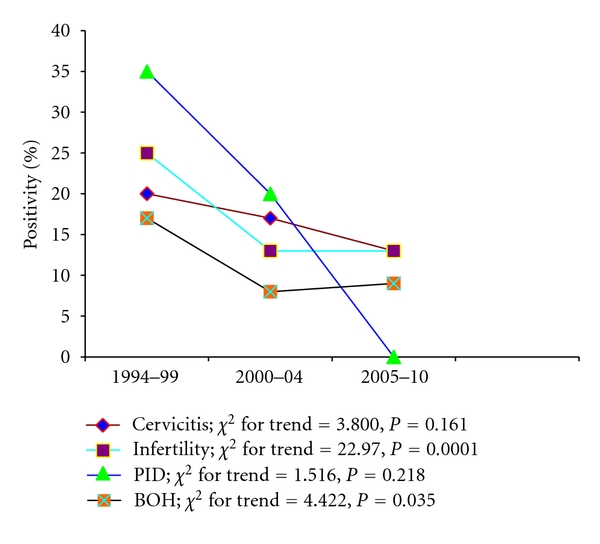
Yearwise positivity rates of Chlamydial infections (based on DFA test results) amongst various disease groups.

In the direct immunofluorescence assay (DFA), the *Chlamydia trachomatis *elementary bodies were visible as regular bright apple green spherical particles. Any smear showing more than 10 such particles were considered positive [12].  Table 1 and Figure 1 depict the results of the DFA tests in the four groups of patients (total 2466) in different block years (1994–99, 2000–2004, and 2005–2010). A total of 391 out of 2466 (15.85%) specimens were positive for *Chlamydia* antigen in DFA test. Amongst the different categories, 73 of 436 (16.74%) women with cervicitis, 268 of 1671 (16.03%) women with infertility, 12 (27.27%) of the 44 women with PID, and 38 of 315 (12.06%) women with adverse pregnancy outcomes (BOH) were antigen positive by DFA test.

As is evident grossly from Figure 1 and Table 1, there seems to be a trend towards a gradual decline in the antigen positivity rates over the years in all the four disease groups. However, statistically significant decline was noted only in the infertility group which showed an overall significant decline in the disease prevalence (Figure 1 and Table 1; *χ* ^2^ trend = 22.97, *P* = 0.0001). A similar trend was also observed when the data were compared between the block years I and II and the block years I and III, for the infertility group only (Table 1).

In tissue culture isolation, bright apple green fluorescent para nuclear *Chlamydial *inclusions were visible in positive specimens. From the 1507 specimens processed for tissue culture isolation, 198 (13.1%) gave positive results; from the same 1507 specimens *Chlamydia* antigen could be detected from 275 (18.2%) specimens. The detailed comparative results between DFA and tissue culture isolation are shown in Table 2.

In the PCR assay for amplification of *C. trachomatis* 517 bp cryptic plasmid gene, 44 out of 333 (13.2%) of the cervical specimens showed positive results; the same 44 specimens were positive for antigen detection by DFA test: both the tests showing complete concordant results in these 333 specimens. 

**Figure 2:**
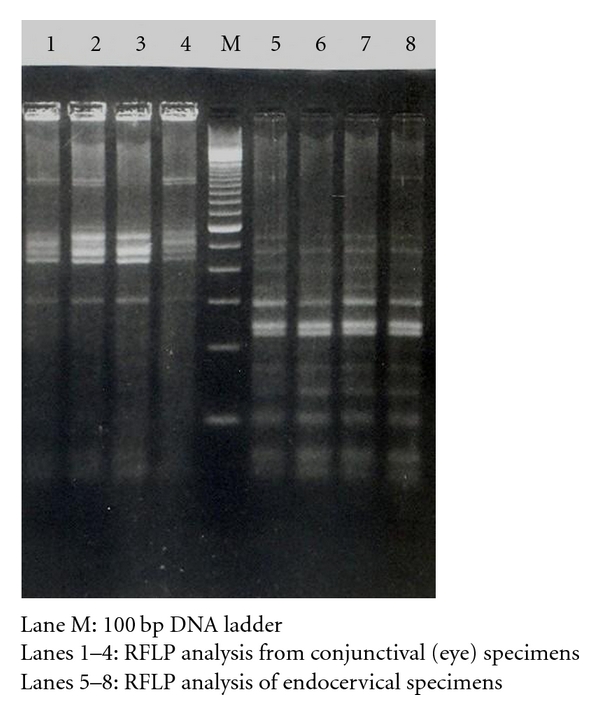
RFLP analysis of the PCR amplified MOMP gene from clinical specimens in 3% agarose gel.

From the 50 endocervical specimens processed for the PCR assay for amplification of the MOMP gene, 22 (44%) were positive. In RFLP pattern analysis, upon examination of the DNA bands in 3% agarose gel stained with ethidium bromide, all the cervical specimens showed a similar pattern, which corresponded to published RFLP pattern of *C. trachomatis* serovar E. For cervical specimens, after complete digestion with Alu1 enzyme, 5 DNA bands were visualized, corresponding to 2 bands each of 100 bp, one band of 138 bp and 2 bands each of 250 bp. (Figure 2).There was a clear-cut distinction between the RFLP patterns of conjunctival and endocervical specimens. The RFLP pattern produced by the conjunctival specimens matched with those of *C. trachomatis* serovar A [15].

## 4. Discussion

In the present study, *Chlamydia* antigen could be detected in 391 out of 2466 (15.85%) of patients studied over a 16-year period, significantly in 27.27% cases with PID and 16.03% cases with cervicitis. (Table 1). Barring a few isolated reports, no such long-term study regarding involvement of *C. trachomatis* in various female genital infections is available from India. According to the isolated reports, *Chlamydia* antigen could be detected in 38.46% of PID patients from Bombay in 1990 [16], in 35.22% of PID patients from Delhi in 1991 [17], in 35.22% patients of PID, and in 42.8% patients of cervicitis from Delhi in 1993 [8]. Results of a few community-based studies spanning 1-2-year period to determine *Chlamydia* prevalence in female genital infections were available. According to these reports, in 2000, *Chlamydia* prevalence was 23.2% in female sex workers attending STD clinics in Bombay [18], *Chlamydia* antigen positivity was 12.2% in women with vaginal discharge (cervicitis) and 28.7% in all symptomatic women in urban slums in Delhi in 2000 and 2001, respectively [19, 20]. Since the current study is based on presence of laboratory proven *Chlamydial *infection among broadly symptomatic women attending hospital during past 16 years, the results are more or less comparable to other reports from different parts of India. Though we presume that studies reporting higher positivity rates might have been conducted on very select group of patients. As was mentioned earlier, in the present study there was a trend towards an apparent decline in the antigen positivity rates over the years, the exact reasons for which are not clear. 

Differentiation of *C. trachomatis* servoars by the use of RFLP pattern analysis of amplified MOMP DNA has been used widely [9, 10, 21, 22]. Such differentiation helps understanding the pathogenesis and epidemiology of the disease. This method has an advantage, as it alone can be used for direct typing of most of the serotypes of *C. trachomatis* [15, 21]. In a study by Sayada et al. [15], 78% of the clinical isolates could be typed directly by using *Alu 1* enzyme alone. In this study we compared the banding pattern of Alu1 digested MOMP gene from the clinical samples with the published pattern in the literature [15] and the analysis showed the presence of *C. trachoamtis* E serovar in the endocervicalspecimens.Poole and Lamont[23] had concluded from a study that serovars E, D, and F were the most commonly found serovars in patients with urogenital infections. We could detect only one genotype, serovar E of *C. trachomatis* in the 22 specimens positive in PCR assay for MOMP gene. Although detection of only one serovar is uncommon, the small number of specimens processed only from cervicitis might have been the reason. Nonetheless, it reveals the most prevalent serovar causing cervicitis.

Although PCR-RFLP is of importance in the epidemiological studies and very little amplified DNA is required, it does not reveal the full extent of sequence variation in some strains [15, 23]. However, the RFLP analysis using the enzyme *Alu 1* is still preferred by many and it can differentiate between most of the serovars of *C. trachomatis* and thus this could be a starting point for further differentiation of the isolates by sequencing techniques [24]. Nevertheless, RFLP performed directly with cervical specimens in the present study, showed that, under optimized reaction conditions, amplification of an approximately 1 kb omp1 fragment followed by digestion with Alu1 enzyme could determine the most prevalent serovar of *C. trachomatis*. This is in agreement with the observations by others [21]. For comparison purposes, we had included a few specimens from *C. trachomatis* eye infections for genotyping, these belonged to serovar A which corroborated with our previous serotyping result [25]. This further strengthens the authenticity of the results of the present study. Although we could detect only *C. trachomatis* serovar E, from India, others have reported presence of servaors D, E, G I, and F, representing 92% of their isolates [8]. In another study from a different part of world, *C. trachomatis* serovars D, E, and F were found to be the most common types by RFLP pattern analysis in 35 specimens from urogenital infections [26].

## 5. Conclusion

Overall *Chlamydia *antigen detection rate varied from 12.2% to 27% in different female genital infection patients (2466) attending hospital over a period of 16 years in New Delhi, India. Even though the exact reason was not clear, there was significant decline and an apparent decline in *Chlamydia* antigen positivity between years 1994–2000 and 2000–10, respectively. In the 333 specimens processed for both antigen detection and PCR assay, equal number of positives (13.2%) was detected with concordant results. PCR-based RFLP pattern analysis concluded the prevalent serovar as *C. trachomatis* serovar E in cervicitis patients. 

##  Conflict of Interests

There is no conflict of interests with any one regarding this study.

## Figures and Tables

**Table 1:** Antigen positivity in patients of various disease groups from 1994 to 2010.

Block-years	Cervicitis (Pos./specimens)	Infertility (Pos./specimens)	PID (Pos./specimens)	BOH with adverse pregnancy outcomes (Pos./specimens)
I-94–99 (684)	21/104 (20.2%)	103/414 (24.9%)	8/23 (34.7%)	24/143 (16.8%)
II-2000–2004 (1011)	36/211 (17.0%)	84/631 (13.3%)	4/20 (20%)	12/149 (8%)
III-2005–2010 (690)	16/121 (13.22%)	81/626 (12.9%)	0/1 (0%)	2/23 (8.7%)
2385	58/355	268/1671	12/44	38/315
	I versus II; *P* = 0.497 I versus III *P* = 0.159 II versus III *P* = 0.354	I versus II; *P* = 0.0001 I versus III *P* = 0.0001 II versus III *P* = 0.910	I versus II; *P* = 0.461 I versus III *P* = 0.717 II versus III *P* = 0.419	I versus II; *P* = 0.036 I versus III *P* = 0.495 II versus III *P* = 0.760
	*χ* ^2^ trend = 1.964	*χ* ^2^ trend = 22.97	*χ* ^2^ trend = 1.516	*χ* ^2^ trend = 4.422
	*P* = 0.161	*P* = 0.0001	*P* = 0.218	*P* = 0.035

**Table 2:** Comparison of DFA with tissue culture isolation.

	Tissue culture		
DFA	Positive (%)	Negative (%)	Total
Positive	108	167	275 (18.24%)
Negative	90	1142	1232
Total	198 (13.13%)	1309	1507

Characteristics of DFA in relation to tissue culture.

Sensitivity = 54.5% (47.3, 61.6), PPV = 39.2% (33.4, 45.3).

Specificity = 87.2% (85.3, 89.0), NPV = 92.7% (91.1, 94.0).

Agreement = 82.9% (80.0, 84.8).

PPV: positive predictive value, NPV: negative predictive value, DFA: direct immunofluorescence assay.
